# Measuring Safety Culture Using an Integrative Approach: The Development of a Comprehensive Conceptual Framework and an Applied Safety Culture Assessment Instrument

**DOI:** 10.3390/ijerph192013602

**Published:** 2022-10-20

**Authors:** Karolien van Nunen, Genserik Reniers, Koen Ponnet

**Affiliations:** 1 Research Chair Vandeputte, University of Antwerp, 2000 Antwerp, Belgium; 2 Safety and Security Science Group, Faculty of Technology, Policy and Management, Delft University of Technology, 2628 BX Delft, The Netherlands; 3 Antwerp Research Group on Safety and Security (ARGoSS), Faculty of Applied Economics, University of Antwerp, 2000 Antwerp, Belgium; 4 Centre for Economics and Corporate Sustainability (CEDON), KU Leuven, 3000 Leuven, Belgium; 5 Research Group for Media, Innovation and Communication Technologies, Department of Communication Sciences, imec-mict Ghent University, 9000 Ghent, Belgium

**Keywords:** safety culture, integrative approach, conceptual framework, assessment

## Abstract

An exponential amount of academic research has been dedicated to the safety culture concept, but still, no consensus has been reached on its definition and content. In general, safety culture research lacks an interdisciplinary approach. Furthermore, although the concept of safety culture is characterised by complexity and multifacetedness, the safety culture concept has been characterised by reductionism, where models and theories simplify the concept in order to better grasp it, leading to confined approaches. In this article, the multifacetedness of safety culture is acknowledged, and the topic is addressed from a safety science perspective, combining insights from multiple academic disciplines. An integrative and comprehensive conceptual framework to assess safety culture in organisations is developed, taking into account the limitations of existing models, as well as the needs of the work field. This conceptual framework is called the ‘Integrated Safety Culture Assessment’ (ISCA), where the ‘assessment’ refers to its practical usability. The practical rendition of ISCA can be used to map the safety culture of an organisation and to formulate recommendations in this regard, with the ultimate goal of bringing about a change towards a positive safety culture. The comprehensiveness of ISCA lies in the inclusion of technological factors, organisational or contextual factors and human factors interacting and interrelating with each other, and in considering both observable or objective safety-related aspects in an organisation, and non-observable or subjective safety-related aspects. When using ISCA, organisational safety culture is assessed in an integrative way by using a variety of research methods involving the entire organisation, and by taking into account the specific context of the organisation.

## 1. Introduction

**Figure 1:**
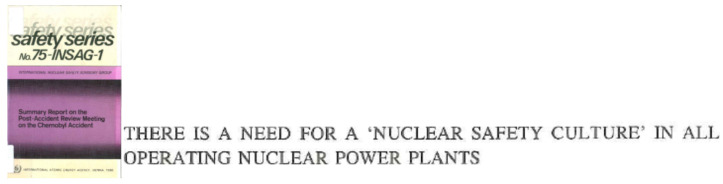
The concept of safety culture was introduced in 1986 in the summary report of the post-accident review meeting on the Chernobyl accident [1] (p. 76) (This cover image and sentence are used with permission from the International Atomic Energy Agency. No further use is allowed).

The concept of safety culture was introduced in 1986 in a summary report of the post-accident review meeting on the Chernobyl accident [1]. This report asserted that “there is a need for a ‘nuclear safety culture’ in all operating nuclear power plants” [1] (p. 76) (see Figure 1). Ever since, an exponential amount of academic research has been dedicated to the safety culture concept [2]. Further, in the work field, safety culture has been a popular topic for many years.

All types of organisations and all organisations sectors are engaged in the topic; the former comprising organisations ranging from small and medium-sized enterprises (SMEs) [3] to multinationals [4,5], the latter including, amongst others, the (petro)chemical sector [6], the nuclear sector [7,8,9] the construction sector [10,11], and the food industry [12]. Consultancy bureaus and public institutions are also invested in this topic by, for instance, offering safety culture improvement programs [13].

Despite this attention from the academic field and the work field to the safety culture concept, no consensus has been reached on its definition and content [14,15]. This lack of consensus, and the abstractedness surrounding safety culture, leads to the concept often being used as an ‘umbrella’, as indicated by Guldenmund [16]. Both the term ‘safety’ and the term ‘culture’ are not straightforward and are characterised by complexity, multifacetedness, and indefinability. As described by Hollnagel [17], there is currently no agreement on what ‘safety’ entails because it does not exist in any tangible or material sense. In 1976, Williams, a researcher in the field of Cultural Studies, described ‘culture’ as one of the most complicated words that exists in a language. Williams indicates that the main cause for this is that the term is used in several distinct intellectual disciplines and in several distinct and incompatible systems of thought [18] (p. 87). This is exactly what is still seen within safety culture research. Anthropology, sociology, engineering, psychology: the concept of safety culture has been the topic of research in a broad range of academic disciplines [5,19]. In addition, in the field of practice, managers, regulators, and consultants are engaged with the topic [20]. The lack of interdisciplinarity and transdisciplinarity between these fields or research causes different approaches, viewpoints, and points of emphasis to add to the multifacetedness already characterising safety culture. The lack of consensus regarding the definition and the content of safety culture inevitably results in a lack of consensus on how safety culture can be measured and altered (and even if safety culture can be measured and altered) [21,22].

The goal of this article is to provide an integrative conceptual framework for organisational safety culture, taking into account the limitations of existing models and the needs of the work field and expounding its practical rendition to the field. This practical rendition can be used to map the safety culture (and sub-cultures) of an organisation, followed by the substantiated formulation and implementation of improvement strategies. 

## 2. Scientific and Practical Relevance 

A large amount of academic research has already been dedicated to the concept of safety culture [2]. It remains, however, relevant to further explore this field, both in terms of theory and practice. This scientific and practical relevance is discussed in the following. 

The concept of ‘safety culture’ is a compound of two terms that are abstract and difficult to grasp. The abstractedness and multifacetedness surrounding the concept of ‘safety culture’—and its application—has led, throughout the years, to the use of ‘safety culture’ as a container-concept to which one can attribute values or attach meanings according to one’s own agenda, requirements, or needs. If employees do not behave in a safe way, it is often ‘the safety culture’ that is to blame, or if a company experiences frequent accidents, it is often ‘the safety culture’ that is considered suboptimal. However, the use of safety culture as a container-concept is distinctive, as, with the introduction of the concept of safety culture, the multifacetedness surrounding safety in organisations is acknowledged [23], whereby safety culture cannot be reduced to something simplistic. This article contributes to the field’s understanding of the complexity of the concept of a safety culture through the development of an integrative model of safety culture. The multifaceted character of safety culture is approached in such a way that safety culture is defined, conceptualised, and visualised in a structured, coherent, and comprehensible way.

Additionally, the integrative model representing safety culture is developed based on scientific insights from various research disciplines. A multitude of approaches and viewpoints exist regarding the interpretation of the concept, but an interdisciplinary and transdisciplinary approach is lacking. This article aims at facilitating the discussion by bringing different points of view together through a truly interdisciplinary lens, where the topic is approached from a safety science perspective, combining insights from multiple academic disciplines, such as sociology, engineering, and psychology. Insights from sociology include, for instance, how social structures and institutions can influence safety. Insights from an engineering focus on the formal and managerial aspects and systems that have an influence on safety (such as management systems, procedures, and policies) [19,24]. In addition, insights from psychology focus on how workers feel about safety and perceive safety, and on their attitudes and behaviours regarding risks and safety [19,24].

The added value of this article for the academic field lies in the integrative and transdisciplinary definition and conceptualisation of safety culture. Furthermore, this article also has an important practical relevance. Besides the multitude of safety theories and models, a vast number of practical tools to measure and improve safety culture is also available. Many organisations show significant interest in and commitment to applying safety culture theories and methods to enhance their organisational safety performance [25]. Of course, this is under the assumption of a good definition and understanding of what constitutes ‘safety culture’. The concept, as already mentioned, is often misunderstood, and erroneous understanding and conceptualising of the concept implies inadequate or inaccurate measuring of the safety culture of an organisation. This could lead to the implementation of improvement strategies that are not aligned with the actual needs of the company, which could even impact safety in a negative way. The applied assessment instrument developed in this article aims at accommodating the limitations of existing tools, and at creating clarity regarding the definition and conceptualisation of the concept by acknowledging the multifacetedness of safety culture by using an integrative approach. Therefore, it is prevented that, in practice, safety culture is reduced to only one or only a few components of the concept, such as behaviours of people or incidents occurring in a company.

Both the conceptual framework and the applied assessment instrument, as explained in this article, are developed in close cooperation and collaboration with the key stakeholders as regards safety culture, namely safety professionals, organisations, and their employees. 

From a scientific point of view, it is important to determine in an integrative and multidisciplinary way the factors constituting the safety culture of organisations. Additionally, from a practice-oriented point of view, it is key that this theory can be converted to a usable instrument to assess the safety culture, and to formulate recommendations in this regard, with the ultimate goal of bringing about a change towards a positive safety culture. When developing the conceptual framework, its applicability in practice is an important point of attention. Safety culture is a complex concept, and this should be acknowledged in the theoretical field, but the concept has to be elaborated in such a way that it is structured, comprehensible, and supported by the target audience the research wants to serve, i.e., safety professionals, organisations, and their employees. By means of involving the work field throughout all stages of the research, an accurate interaction between theory and practice is ensured. The close cooperation with the work field implies that the needs of the work field are taken into account, and that the research output has added value for organisations and their employees.

## 3. Definition of Safety Culture

In this article, safety culture is defined as the following:

The safety culture of an organisation reflects the broad spectrum of established safety-related human, organisational or contextual, and technological aspects prevailing in the entire organisation. It entails observable, tangible factors, being the safety management of an organisation, the physical working environment, and how individuals behave in relation to safety. In addition, it entails non-observable, less tangible factors, being the values and attitude of individuals in relation to safety, and the shared perceptions of safety. All these safety-related aspects interact with each other in a dynamic way. Differences within an organisation can manifest themselves into different sub-cultures.

The rationale behind this definition originates from both experiences in the work field and commonalities among safety culture definitions that can be found in the literature [13,26]. Firstly, let us turn to experiences from the work field. Due to the complexity of safety culture, a simplified conceptualisation is inapposite and will lead to a distorted picture of reality. Previous research has shown that models, and the practical tools derived from these models, tend to only address a subset of the concept of safety culture without adhering to a comprehensive viewpoint and approach [13]. To adhere to this need for a comprehensive viewpoint and approach, a broad spectrum of safety-related aspects is included in the definition, which takes into account human aspects, organisational or contextual aspects, and technological aspects interacting and interrelating with each other. Safety culture assessments often place the main emphasis on human behaviour, while diminishing the importance of other human aspects, such as the safety values and attitude, and the organisational and technological aspects prevalent in an organization [13].

Aside from experiences from the work field, the used definition originates in commonalities among safety culture definitions abounded in the safety literature [27]. When disentangling the different parts mentioned in the definition above, the ‘broad spectrum of safety-related aspects’ refers to the multidimensionality of the concept of safety culture [5,16,28]. The broad spectrum also means the inclusion of both type I-safety (which addresses possible accidents with a high probability and a low impact) and type II-safety (which addresses possible accidents with a low probability and a high impact). The trichotomy of ‘human, organisational and technological aspects’ is assumed as a fundamental safety principle [29]. The trichotomy is based on systems view and emphasizes interactions and interdependencies between the three aspects [30], and it is also seen as an approach to understanding complex systems [31]. The ‘established’ aspects refer to the characteristics of a safety culture (or the broader organisational culture) as being relatively stable and resistant to change [16,27,32]. A recurrent characteristic in the academic literature is that a safety culture is shared between people [16,33,34]. The ‘entire organisation’ emphasizes the contribution to the safety culture of an organisation by everyone, at all levels of the organisation [32]. The ‘interaction’ refers to the general assumption that several interacting elements or factors form the safety culture of an organisation [35,36]. The ‘different sub-cultures’ reflect the assumption that results of a safety culture measurement may differ within a single organisation between, for instance, departments or positions [16]. 

The given definition of organisational safety culture only reflects internal safety aspects. However, organisations exist within a broader context that also has its impact on the organisational culture in general, and the safety culture in particular [19,37]. For instance, Yorio et al. [38] advocate in their article on safety culture across cultures that the national culture also needs to be integrated into organisational safety culture theory. Besides the national culture, external factors entail the political situation, the socio-economic status, the level of technological development of a country or a region, and the local prevailing (safety) policies, regulations, and legislations [19,39]. For instance, prevailing safety regulations can affect the standards to which the physical workplace has been built [37]. Further, the COVID-19 pandemic has affected the safety culture of an organisation. For instance, research has shown that during the COVID-19 pandemic, reductions in incident reporting could be seen [40]. These changes were not only due to changes in the volume or nature of work but were also caused by changes in risk perception [40]. In the safety culture framework expounded in this paper, this broader context and external factors are not included. Although it is acknowledged that external factors can significantly influence a safety culture of an organisation, a single organisation does not have the ability to alter such external factors. As indicated by Øien et al. [41], it is recommended to concentrate on changeable and controllable factors when addressing the safety of an organisation. 

## 4. An Integrative Conceptual Framework of Safety Culture

The foundation of this integrative conceptual framework of safety culture lies in the model developed by Vierendeels et al. [26]. Based on an extensive literature review regarding existing studies and models with respect to safety, Vierendeels et al. [26] developed an overall conceptual ‘big picture’ model of safety culture. This model is called The Egg Aggregated Model (TEAM), and the added value of TEAM lies in its integrative and holistic viewpoint and approach. In this paper, the merely theoretical viewpoint and approach of TEAM will be translated into a conceptual framework that is useable in the work field. This conceptual framework is called the Integrated Safety Culture Assessment, or abbreviated ISCA, where the ‘assessment’ refers to its practical usability.

TEAM consists of three major building blocks or domains interacting with each other in a cyclic and reciprocal way: a human, an organisational or contextual, and a technological domain [21,42]. Besides these three building blocks, an important dichotomy in TEAM is the distinction between observable and non-observable factors [26]. These premises are used as the basis of ISCA, where in this framework, safety culture consists of:

- Observable safety-related aspects are tangible, visible, and can be observed in an organisation. It concerns what people do, such as the performed safety behaviour, and what the organisation has, such as the safety policies that can be consulted in company documents [35]. It entails all objective safety-related aspects of an organisation.
- Non-observable safety-related aspects are less tangible, less visible, and not directly observable within an organisation. It concerns all subjective safety-related aspects, for instance, what employees think regarding safety in the company, or the attitude they have towards safety.

This dichotomy can be applied to the three building blocks of the safety culture framework; the human, the organisational or contextual, and the technological domain:

- The technological domain entails all safety-related aspects regarding the physical working environment and how these characteristics are taken into account to manage safety in the organisation. The physical working environment is determined by the main activities of an organisation, the associated risks and possible accident scenarios, and the corresponding safety measures. The technologies, processes, design, materials, equipment, etc., used in an organisation are decisive for the risks and possible accident scenarios present in an organisation. This domain only consists of observable aspects.
- An organisational or contextual domain comprises all safety-related aspects on a company level. It entails observable organisational aspects reflecting the safety management of an organisation (e.g., resources for safety such as money and people and safety policies). In addition, it entails non-observable organisational aspects reflecting the perceptions of how the organisation deals with safety. It refers to the (internal) safety context that is created by decisions on a company level.
- A human domain comprises all safety-related aspects on an individual level. It entails observable human aspects being the safe and unsafe behaviours of individuals, and it entails non-observable human aspects such as safety competence or personal safety priorities of the individuals in the organisation.

**Figure 2:**
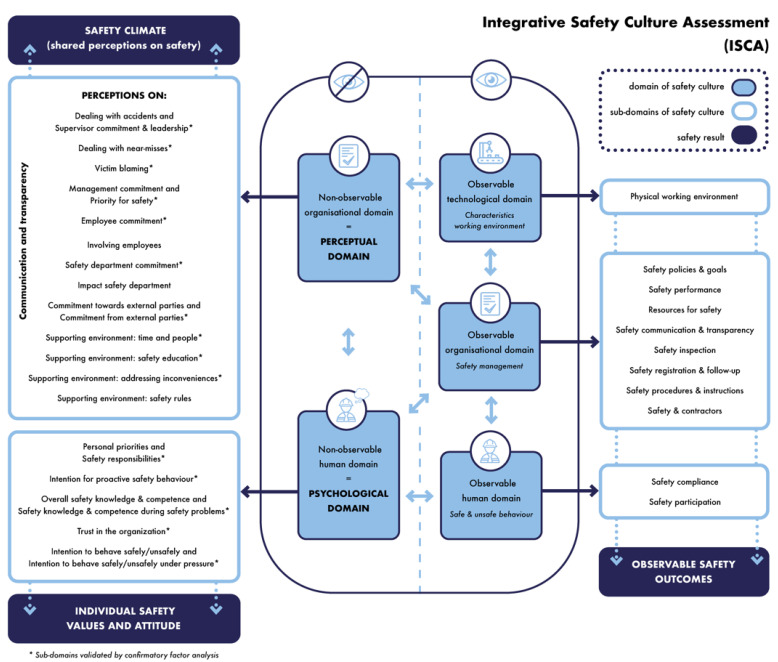
Integrative Safety Culture Assessment (ISCA).

This structure leads to five domains which together form the safety culture of an organisation, as can be seen in the centre of Figure 2. The two-way arrows in Figure 2 indicate the dynamic interactions between the different domains. For instance, safety behaviour is not a standalone domain; the way people act (and interact) should always be seen in the organisational and technological context of the organisation. Changes in the organisational and/or technological context will, most of the time, also lead to changes in the actual behaviour, which reflects the cyclic and reciprocal interaction of the different domains of safety culture.

The next step is to make the transition from these five abstract, holistic domains, to measurable safety outcomes or safety results. In the next parts of this article, this practical transition will be explained for the non-observable domains and the observable domains, respectively.

## 5. Assessing the Safety Culture

**Figure 3:**
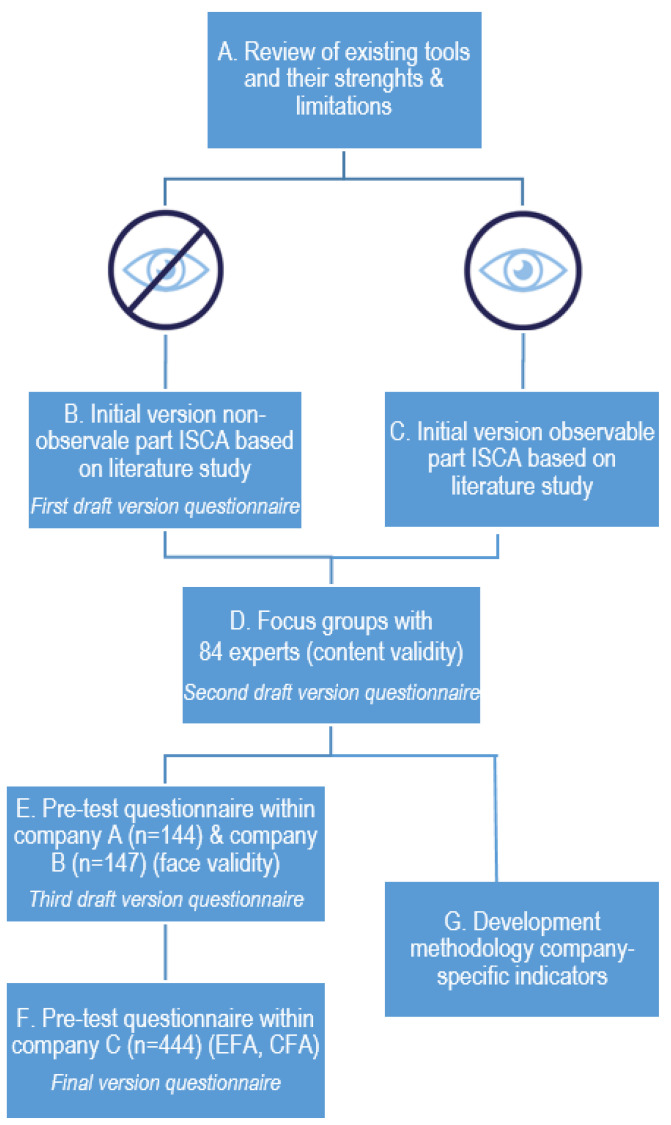
Development process of Integrated Safety Culture Assessment (ISCA).

To translate the five domains of safety culture into measurable safety results, the holistic domains need a further breakdown into measurable sub-domains. Figure 3 shows the approach to defining the sub-domains, how the methodology of ISCA was determined, and how the usability of ISCA in the work field was ensured.

First, a literature review of existing tools to measure and improve safety culture was conducted to identify current strengths and limitations (see step A Figure 3) [13]. Based on this review, the following recommendations for developing ISCA were made:

- When assessing the safety culture of an organisation, an integrative viewpoint and approach must be used where human, organisational or contextual, and technological factors must be taken into account.
- The involvement of the entire organisation is crucial. All layers of the organisation must be included when assessing safety culture: the safety department, employees, supervisors, management, and external parties such as contractors.
- The assessment needs to take into account the specific needs and context of an organisation. Developing a tool that can be applied to all sectors and sizes of organisations, a so-called ‘one size fits all tool’, is not feasible and not apposite.
- A variety of methodologies must be used when diagnosing the safety culture of an organisation. Each methodology has its limitations, and these limitations can be reduced by applying data triangulation.

Based on these recommendations, the overall framework of ISCA was determined: the safety culture of an organisation must be assessed in an integrative way, by using a variety of research methods where the entire organisation is involved and where the assessment takes into account the specific context of the organisation.

### 5.1. Steps in the Assessment of Safety Culture (ISCA-Approach)

**Figure 4:**
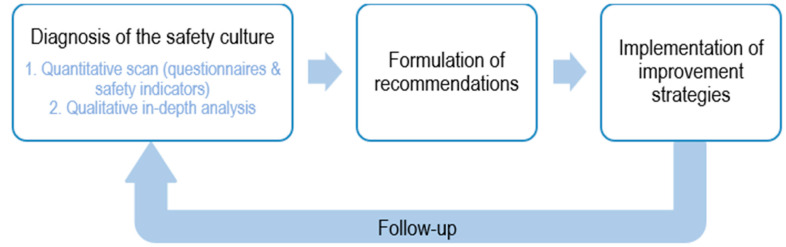
ISCA-approach—steps in the assessment of the safety culture.

Before the sub-domains were defined, decisions were made regarding the methodology of ISCA. Assessing safety culture takes place in different steps (see Figure 4). First, a measurement or ‘diagnosis’ of the safety culture is performed. Secondly, based on the diagnosis of the safety culture, improvement strategies are formulated, and priorities are set, followed by the implementation of the formulated recommendations. The key to this approach is follow-up. Continuous attention to safety culture is fundamental in order to optimise (and maintain) a safety culture.

Diagnosing the safety culture can be further breakdown into different steps. First, a quantitative scan is performed. Depending on the specifics of a domain (i.e., observable or non-observable), the measurement methodology of this quantitative scan varies. The non-observable domains represent the subjective safety-related aspects and can be mapped in this first phase by means of questionnaires (see Section 5.2). The observable domains represent the objective safety-related aspects and can be mapped in this first phase by means of safety indicators. Input for these safety indicators is derived from document analysis, data analysis, and observations in the organisation (see Section 5.3). 

After the quantitative scan, the diagnosis of the safety culture is complemented with an in-depth qualitative analysis, where both the observable and non-observable domains can be explored more in-depth by means of interviews and/or focus groups (see Section 5.5). This in-depth qualitative analysis is important in order to map the specific causes and possible nuances of the safety culture outcomes as diagnosed in the quantitative scan.

### 5.2. Assessing the Non-Observable Safety Culture Domains

A questionnaire is a well-suited methodology for mapping or diagnosing the non-observable safety culture domains, as these domains represent the subjective safety-related aspects of an organisation. As shown in Figure 2, both the non-observable organisational domain and the non-observable human domain comprise several sub-domains. These sub-domains represent the constructs used in the questionnaire to map the non-observable domains. As mentioned by Anrijs et al. [43], assuring the accuracy of the examined constructs is key when conducting questionnaire research. To ensure that the used constructs and associated items are valid and reliable, a multi-phase method was performed. In the first phase, constructs and items were generated based on a literature study (see step B Figure 3), and the content validity was evaluated by a large team of experts (see step D Figure 3). The second phase was the testing phase. The first test within two companies resulted in 291 combined respondents, and was used to evaluate the face validity (see step E Figure 3). A second test resulted in 444 respondents. An exploratory factor analysis (EFA) and a confirmatory factor analysis (CFA) were carried out (see step F Figure 3), after which the final version of the questionnaire was established. 

#### 5.2.1. Phase 1. Construct and Item Generation: Literature Study and Content Validity

Literature study

The non-observable part of the safety culture model firstly consists of an organisational domain or perceptual domain. This domain reflects the shared perceptions of safety or the safety climate of an organization [26]. Safety climate is a multidimensional construct that encompasses a wide range of individual evaluations or perceptions of the value of safety in the work environment [44,45]. It encompasses shared perceptions of the safety context that is created by decisions on a company level. Secondly, the non-observable part of the safety culture model consists of a human domain or psychological domain, which represents individual safety values and attitudes [26]. The distinction between the perceptual domain and the psychological domain reflects itself in the distinction between how an individual (or groups of individuals) thinks about or perceives safety within the organisation (perceptual domain) versus how an individual evaluates their own safety values and attitudes (psychological domain). For instance, the sub-domain ‘priority for safety’ in the perceptual domain reflects how someone perceives the priority that the organisation gives to safety. In the psychological domain, ‘priority for safety’ reflects the priority that the individual person gives to safety.

A literature study (see step B Figure 3) on safety culture, safety climate, safety perceptions, and safety attitudes was performed. Based on this literature study, key constructs or sub-domains and their corresponding items (i.e., the questions constituting the questionnaires) were considered for the non-observable parts of the safety culture model (perceptual domain and psychological domain). 

The first draft of the ISCA questionnaire based on the literature study consisted of 99 items divided over 16 sub-domains. Of these items, 69 consisted of positively worded statements, and 30 consisted of negatively worded statements. Besides the items, four standard (demographic) characteristics of respondents are also questioned: age, the highest level of education achieved, seniority, and position at the company. If relevant for the specific company under investigation, additional characteristics can be questioned, such as working time present on site.

One of the recommendations deriving from the review of existing tools (see step A in Figure 3) is to take into account the specific needs and context of an organisation when assessing safety culture. To assure validity and reliability, the items and constructs of a questionnaire should be stable. However, two types of alterations can be made to the questionnaire in order to take into account the specifics of an organisation. Firstly, terms that are used in the questionnaire are tailored to the common vocabulary in the organisation (e.g., supervisor, N+1, team leader, direct manager,…). In addition, the name of the organisation is incorporated in some of the questions, such as ‘At [name of the company], I am involved in making the workplace safer’. Secondly, questions that are not applicable to the entire organisation or to specific departments are not questioned for the entire organisation. For instance, if the organisation does not work with personal protective equipment (PPE), questions on PPE are omitted. This approach is used to optimise the familiarisation of the questionnaire for the respondents.

Content validity

As a next step, the first draft version of the ISCA questionnaire was evaluated on content validity (see step D Figure 3). Content validity is an important step when developing a questionnaire, as it assesses the relevance and added value of all items in relation to the constructs to which the items belong [43,46,47]. Content validity also evaluates the clarity and parsimony of item wording [43,46]. The content validity of the ISCA questionnaire was assessed in a qualitative way by 84 experts in total. During five focus groups, which each took one and a half to two hours, the experts discussed in groups of three to five on the wording, grammar, item allocation, and constructs of the ISCA questionnaire. The Dutch and Belgian experts were mainly professionals in the field, i.e., safety managers and safety specialists from a diverse range of organisations. Experts had experience in a variety of sectors (e.g., construction, transportation, production, retail, and chemical industry) and in a variety of company sizes (from SMEs to large multinationals). Further, five academic researchers specialised in organisational safety were part of the expert pool. 

Based on the feedback of the experts, several changes were made. An important adjustment that was made was the differentiation of the items depending on the position of the respondent. This is also in line with one of the recommendations deriving from the review of existing tools (see step A in Figure 3) to take into account all different layers of the organisation when assessing safety culture. Items were added, removed, or adjusted based on the position of the respondent. For instance, for respondents with a supervising position or a management position, the following question was added: ‘The people whom I supervise attach a great deal of importance to working safely’. A question that was only applicable to employees, is, for example, the following: ‘Sometimes I do not observe the safety rules due to pressure from my direct supervisor’. The following positions are distinguished (If someone has a combined position, for instance, a supervising position combined with the position of safety manager, the respondent is asked to choose the position in which most of the working time is spent. Especially in smaller organisations, this is common):

(i) Employees: these are the people responsible for carrying out the organisation’s core activities (e.g., production in a manufacturing environment or performing transport in a transportation company) and the supporting activities (e.g., administration or maintenance). It concerns the ‘operational staff’. Employees have no or very limited supervision over others within the organisation.
(ii) Supervisors: these are the people responsible for the day-to-day management of the organisation’s core activities and supporting activities. Supervisors are directly in charge of one or more employees. Usually, supervisors are regularly present and active on the work floor.
(iii) Management: these are the people occupying the highest positions within the organisation (or within one location of the organisation if there are multiple locations). They are in charge of the overall management of the organisation and they are usually less present and active on the work floor.
(iv) Employee in the safety department (with or without supervising position).
(v) External parties, i.e., contractors (if applicable), and customers or clients (if they are regularly present on site of the organisation).

According to the experts, the distinction between the perceptual domain, on the one hand, and the psychological domain, on the other hand, should be represented more clearly. This was accommodated by formulating the questions probing for the shared perceptions on safety in a more general way (e.g., ‘At this company…’, ‘The employees…’, etc.). Questions probing for the individual safety values and attitudes were formulated with the respondent as the subject of the statement (e.g., ‘If a task is too unsafe, I refuse to do it’). By making this division between ‘they’ and ‘I’ in the questioning format, the distinction between the perceptual domain and the psychological domain, respectively, is more explicit.

Furthermore, the wording of some of the negatively worded statements was reformulated in the same positive direction as other items (e.g., ‘When work is running behind schedule, safety rules may not be observed’ was changed into ‘The safety rules are observed, even when work is running behind schedule’). Several experts indicated that switching too much between negatively and positively worded statements is confusing and can lead to misinterpretations by respondents [48]. Furthermore, some questions were omitted. For instance, the question ‘Unsafe behaviours of colleagues sometimes puts other workers at risk’ was deleted because this is too obvious, and ceiling answering effects can be expected. Further, the question ‘Equipment, machines and installations are dated’ was deleted because dated does not necessarily imply that it does not function properly. According to the experts, some relevant aspects were missing, which were added to the questionnaire (e.g., ‘After an accident, employees are involved in searching for solutions’, ‘Everyone works safely at [name of the company], even when no one else is around’). Some items were reformulated in order to better capture the essence. For instance, the question ‘At [name of the company], there is regular noise nuisance’ was changed to ‘At [name of the company], noise nuisance is addressed adequately’. After all, it is not necessarily a problem if a regular noise nuisance is present at a company, but it is a problem if the present noise nuisance is not addressed adequately.

After consulting the expert panel, the second draft version of the ISCA questionnaire consisted of 188 items. Questions containing a reference to a specific function or position only apply to the corresponding position. For instance, questions containing ‘The people whom I supervise…’ are only applicable to respondents with a supervising position. Of all 188 items, 128 items were applicable to employees, 147 items to supervisors, 147 items to managers, 133 items to staff members of the safety department, and 34 items to external parties.

#### 5.2.2. Phase 2. Testing Phase: Face Validity, EFA, CFA

Face validity (company A and company B)

As a next step, the face validity of the ISCA questionnaire was assessed in a qualitative way by means of a cross-sectional study with the target group of the ISCA questionnaire (see step E Figure 3). The second draft version of the ISCA questionnaire was administered in company A and company B. Company A is a large chemical company (multinational) located in Belgium. Only staff members of supporting departments, and not of operating departments, participated. The supporting departments concerned the financial department, and facilities responsible for site security, cleaning, electricity maintenance, and food service on-site. Company B is a large chemical company (multinational) located in The Netherlands. In company A, 144 respondents completed the questionnaire, and in company B, 147 respondents. Table 1 (company A and B) presents the descriptive characteristics of the respondents.

Respondents were asked to complete the questionnaire and to evaluate each item of the questionnaire on ambiguity and difficulty in replying. Informed consent was obtained from all respondents before completing the questionnaire, and all data were analysed anonymously.

When administering the questionnaire, items were randomised. Based on the participants’ written feedback, the first adjustment was to cluster the questions according to the topic. All questions on priority for safety were clustered, all questions on dealing with accidents were clustered, and so on. Further, the questions probing the perceptual domain and the questions probing the psychological domain were clustered in order to reduce the cognitive overload for the respondents. 

As mentioned before, questions that are not applicable to the entire organisation or for specific departments are not questioned in that specific organisation or in those specific departments, in order to optimise the familiarisation of the questionnaire for the respondents. However, some respondents were unable to score some of the statements due to non-relevance or unfamiliarity with the topic. Not having the option to indicate ‘not applicable’ could unbalance the actual scores of the statements. Therefore, as the second adjustment, the possibility to answer ‘not applicable’ was added. Even though adding this ‘non applicable’ answer possibility leads to deficiencies in data analysis due to more missing values, the accuracy of the answers of the respondents was considered paramount.

Twelve items were omitted because of overlap with other items (e.g., “[Top management] is concerned with the safety of employees” was deleted because its familiarity with “At [name of the company], [top management] attaches a great deal of importance to the safety of employees”). No items were added. 

This led to the third draft version of the questionnaire. This version includes a total of 87 items (79 items for employees, 71 items for supervisors, 72 items for managers, 62 items for staff members of the safety department, and 38 items for external parties). 

Exploratory factor analysis (EFA) (company C)

The third draft version of the questionnaire was administered within a large chemical company (multinational) located in Belgium, leading to 444 respondents (see step F Figure 3). Table 1 (company C) presents the descriptive characteristics of the respondents. The data from company C are used for exploratory factor analysis (EFA) to assess construct validity.

Given the structure of companies, the number of employees or operational staff is logically higher compared to the number of supervisors, managers, and staff members of the safety department. This also applies to the company under investigation (company C), where 61.0% (n = 271) belongs to the operational staff, 23.4% (n = 104) has a supervising position, 10.8% (n = 48) a management position, and where 1.4% (n = 6) is a staff member of the safety department (Table 1 company C). As different items are applicable to different positions, and the respondent numbers of supervisors, managers, and safety staff members are too low, the items only applicable to the supervising positions, management positions, and safety positions are not included in the factor analysis. In other words, only those items applicable to employees (79 out of the 87 items) were used to perform the EFA. This does not imply that respondents with a non-employee position are deleted from the analysis, as some of the questions are applicable to all positions.

Of the 444 respondents, 381 participants (85.8%) showed missing responses to at least one variable. In terms of all the cells present in the data, 87.0% of them were filled. In order to perform an EFA (and in a later stage, a CFA) with a complete dataset, which is a requirement for the maximum-likelihood method, the expectation-maximization algorithm was performed to impute values on the missing cases. After this technique was employed, the resulting sample size for all variables was 444.

Analysis method EFA. Because the set of items represents two different domains of safety culture, i.e., the perceptual domain on the one hand, and the psychological domain, on the other hand, the EFA was conducted separately for on the one hand items belonging to the perceptual domain (50 out of the 79 items) and on the other hand items belonging to the psychological domain (29 out of the 79 items). Analysing items belonging to these different domains together, inevitably would lead to unrelated variables. 

The maximum-likelihood method was performed on each domain to examine the factor structure underlying the data. Two assumptions were tested before proceeding to the analysis: the sampling adequacy and the test of sphericity. To assess the adequacy of the sample for the factor analysis, the Kaiser–Meyer–Olkin (KMO) measure was consulted. KMO values of 0.60 or higher indicate an acceptable sample, and values between 0.80 and 1 indicate an adequate sample [43,49]. Bartlett’s test of sphericity tests the hypothesis of an unrelated correlation matrix, which is unsuitable for factor analysis as no structure could be detected in such a case. *p*-values of less than 0.05 indicate that factor analysis is useful for applying to the data [43,50].

A possible number of factors on the data structure was examined using the ‘Eigenvalue higher than 1’ criteria as suggested by Hair et al. [51]. Although factor loadings of 0.30 to 0.40 are minimally acceptable, values greater than 0.50 are generally considered necessary for practical significance [51]. Next, coefficients were examined to identify items with cross-loadings (loadings on more than one factor). If one item presented loadings higher than 0.4 on more than one factor, this item was excluded. Additionally, if the item showed a substantially higher loading on any secondary factor, meaning that the item is not measuring the same concept as the other items, it was also excluded. After excluding items (when appropriate), the procedure was executed again. The proportion of the total variance explained by the retained factors is acceptable when it is at least 50% [52]. The Cronbach’s alpha coefficient of each scale should be 0.7 or higher [53]. The EFA was performed using SPSS 25.

Results EFA—Perceptual domain. The examination of the eigenvalues of the first non-rotated solution indicated that any solution from 6 to 10 factors would be feasible. Since a 10-factor solution would make more theoretical sense, this solution was generated using Varimax rotation, and an iterative process of one-by-one dropping items and re-running the analysis took place following two criteria: (1) if an item showed no factor loading above 0.4, and (2) if an item showed cross-loadings of more than 0.4 on 2 or more factors. Four solutions were iteratively generated until arriving at a final solution which is shown in Table 2. This solution has passed the test of sphericity (*p* < 0.001) and sampling adequacy (KMO = 0.882). The total variance retained by the solution is 74.4%.

A subjective assessment of the solution detected that item ‘Id. 1307’ should be excluded due to the theoretical illogical of being part of Factor 1. The low factor loading for this item (λ = 0.401) corroborated this idea. The rest of the factor structure made theoretical sense. Thus, the factor structure presented above will be transferred to the CFA procedure (without item Id. 1307). The minimum number of items per factor is two and the maximum amount of items per factor is eight.

Results EFA—Psychological domain. The process was repeated for the psychological domain. Eigenvalues of an initial non-rotated solution indicated that any solution from 5 to 8 factors would be adequate (eigenvalues around 1). After five iterations following the exclusion criteria presented earlier, 17 items were kept (from the original 29) to form the final factor structure (Table 3). The solution passed the test of sphericity (*p* < 0.001) and sampling adequacy (KMO = 0.847). The constructs jointly accounted for 69.3% of the observed variance. The minimum number of items per factor is two and the maximum amount of items per factor is five.

Confirmatory factor analysis (CFA) (company C)

In the next step, construct validity was tested. Construct validity is defined as “the extent to which a set of measured variables actually represent the theoretical latent construct they are designed to measure” [51]. Confirmatory factor analysis (CFA) was used in the analysis, as it is an adequate method to be used as evidence of the construct validity of theory-based instruments [54]. For the CFA, the dataset from company C (n = 444) was used.

Analysis method CFA. CFA was conducted using SPSS AMOS software, which uses a maximum likelihood (ML) algorithm to estimate the results. ML is the most common method used to estimate parameters in CFA, because of its attractive statistical properties (i.e., asymptotic unbiasedness, normality, consistency, and maximal efficiency) [54]. After defining the model in the software and executing the analysis, four main phases were conducted to examine construct validity (1) assessment of model fit, (2) assessment of convergent validity, (3) assessment of discriminant validity, and (4) respecification of the model (if necessary). The statistics used to assess model fit and their rules of thumb are the following: normed chi-square (χ^2^/df) (the division between the chi-square value and the model’s degrees of freedom) should be less than 4, root mean square error of approximation RMSEA < 0.08, comparative fit index CFI > 0.90, normed fit index NFI > 0.85 [55].

After the assessment of model fit, convergent and discriminant validity were examined. Convergent validity refers to the “extent to which indicators of a specific construct converge or share a high proportion of variance in common” [51]. Standardized factor loading (λ) estimates should be 0.5 or higher and ideally 0.7 or higher. Average variance extracted (AVE) of 0.5 or higher is a good rule of thumb, suggesting adequate convergence. AVE represents the amount of variance that is captured by a construct in relation to the amount of variance due to measurement error. Discriminant validity is defined as the “extent to which a construct is truly distinct from other constructs both in terms of how much it correlates with other constructs and how distinctly measured variables represent only this single construct” [51]. The squared variance extracted estimates for a construct should be greater than the correlation estimates between this and other constructs (squared AVE > p) [56]. A second important concept to test is the construct’s reliability. This was performed using the composite reliability index (which is based on factor loadings) (0.7 or higher suggests good reliability) and Cronbach’s Alpha (which is based on correlations) (0.7 is the minimum acceptable level) [57].

Results CFA—Perceptual domain. *Convergent Validity and Reliability*. The first solution, derived from the EFA, showed AVE lower than 0.500 for Factor 1 (AVE = 0.466) and Factor 7 (AVE = 0.462). The item with the lowest factor loading for each factor was deleted, and the analysis was executed again. The deleted items were ‘Id.1297’ for factor 1 (λ = 0.635) and ‘Id.1342’ for factor 7 (λ = 0.635). The second solution, without those items, also showed AVEs lower than 0.500 for factor 1 (AVE = 0.478) and factor 7 (AVE = 0.488). Following the same logic of the first iteration, items ‘Id.1298’ (λ = 0.615) and ‘Id.1341’ (λ = 0.687) were dropped. The third solution showed acceptable convergent validity according to AVE and good reliability for most factors according to the composite reliability and alpha coefficients (higher than 0.7). The final solution, with indicators of convergent validity and reliability, is shown in Table 4, along with the labels created for each factor. The factor ‘Supporting environment: addressing inconveniences’ (Factor 7) still showed coefficients slightly below acceptable, but since there were no more items to be dropped (only two items left), this factor was kept in the final structure as it is.

The final model presented in Table 5 showed an acceptable fit (χ^2^ (279, n = 444) = 979.801; *p* < 0.001; χ^2^/df = 3.512; RMSEA = 0.075; CFI = 0.902; NFI = 0.870).

*Discriminant Validity*. After determining convergent validity, the discriminant validity was assessed. Table 5 shows the correlations among constructs obtained through CFA. The only factor that lacked discriminant validity (squared AVE lower than one or more correlations) was ‘Management commitment and priority for safety’, which correlates quite strongly (>0.80) with ‘Dealing with accidents and supervisor commitment and leadership’. All other constructs showed good discriminant validity.

Results CFA—Psychological domain. *Convergent Validity and Reliability*. AVE indices of the first solution were not acceptable for Factor 1 and Factor 3. Thus, items ‘Id.1379’ (λ = 0.551) and ‘Id.1355’ (λ = 0.568) were dropped. A second solution still showed unacceptable convergent validity for Factor 1, and item ‘Id.1377’ (λ = 0.648) was dropped. The third solution reached sufficient convergent validity and reliability (Table 6).

*Discriminant Validity*. The solution showed good discriminant validity, as shown in Table 7. All squared values of AVE are higher than inter-construct correlations.

The goodness-of-fit indices for the final model (χ^2^ (67, N = 444) = 297.099; *p* < 0.001; χ^2^/df = 4.434; RMSEA = 0.088; CFI = 0.917; NFI = 0.897) indicate an RMSEA slightly higher than the acceptable threshold of 0.080. A standardized root mean square residual (SRMR), which is an alternative to RMSEA as an indicator of models’ parsimony, showed evidence of good fit (SRMR = 0.058) (values lower than 0.08 are acceptable) [51].

To close this testing phase consisting of face validity, EFA, and CFA, it should be remarked that the items only applicable to the supervising positions, management positions, and safety positions are not included in the EFA and subsequent CFA. This implies that not all of the sub-domains belonging to the non-observable domain are validated by means of EFA and CFA. The non-observable sub-domains validated by EFA and CFA are indicated with an asterisk (*) in Figure 2 and Table 8. The part where the face validity was tested, however, it did include all items applicable to all different positions.

An overview of all non-observable sub-domains and their definitions, supporting literature for each sub-domain as found during the literature study (see step B Figure 3), and an example question belonging to each sub-domain can be found in Table 8 (Due to intellectual property rights, the entire list of questions cannot be disclosed in this article. However, with the purpose of continued scientific research, the questionnaires can be requested to the first author of this article via e-mail). 

### 5.3. Assessing the Observable Safety Culture Domains

Where the non-observable safety culture domains represent the subjective safety-related aspects of an organisation, the observable safety culture domains represent the objective safety-related aspects. A suitable method to map or measure these objective safety aspects, is the use of safety indicators. Objective safety indicators represent data that are free of subjective bias. In other words, objective safety indicators do not reflect an opinion, a perception, or an evaluation of safety aspects [126]. The objectivity of these safety measures implies that the needed data are available within the company without conducting additional research, such as a questionnaire to map the perceptions of employees.

As seen in Section 5.2, the sub-domains of the non-observable part of safety culture can be operationalised by a standardised set of questions. However, the operationalisation of the sub-domains of the observable part of safety culture cannot be standardised, as safety indicators should be tailored to the specific context of the organisation [13]. It is, for instance, very difficult to compose a set of general indicators to assess the level of safety compliance, as this compliance depends on the required behaviour defined by the specific working environment of an organisation. This implies that for the observable safety culture domains, the different sub-domains are fixed, but the operationalisation of these sub-domains is not.

To define the different sub-domains belonging to the observable domains of safety culture, a two-phased method was performed. Firstly, a literature study was conducted (see step C Figure 3), and secondly, the content validity of the defined sub-domains was evaluated by a large team of experts (see step D Figure 3) (see Section 5.3.1).

Subsequently, because the operationalisation of the sub-domains cannot be standardised, but as this operationalisation is always company-specific, a methodology has been developed that enables organisations to compose a set of company-specific safety indicators. This methodology is summarised in Section 5.3.2.

#### 5.3.1. Observable Safety Culture Sub-Domains: Literature study & Content Validity

Literature study

The observable part of the safety culture model consists of three domains. Firstly, the observable technological domain reflects the characteristics of the working environment, and how these characteristics are taken into account to manage safety in the organisation (such as purchasing and maintenance of production installations). It looks into the main activities of an organisation, the associated risks and possible accident scenarios, and the corresponding safety measures. Secondly, the observable organisational domain reflects the safety management of an organisation, which can be seen as the formalised (and therefore documented) system of controlling risks within an organisation [21], and it reflects all the supporting processes to effectuate a safety management system [19]. In other words, the observable organisational domain includes those safety aspects which entail a formal decision within the company (mostly on a higher hierarchical level). Thirdly, the observable human domain reflects the individual behavioural aspects with regard to safety.

An initial version of the sub-domains belonging to the observable part of safety culture was developed based on a literature study on safety culture, risk assessment, safety management systems, safety behaviour, safety performance, and safety performance indicators (see step C Figure 3). A set of examples of possible safety indicators was also composed for each sub-domain.

Content validity

This initial version of sub-domains and safety indicator examples was evaluated on content validity by 84 experts during five workshops (see step D Figure 3). Based on the insights of the experts, some adjustments and refinements were made to the descriptions of the sub-domains (Table 9), and some overall recommendations were formulated regarding the composition of the company-specific indicators. Examples of these recommendations are:

- Take into account quantities of safety measures, and also look at the quality of these safety measures (e.g., percentage of planned safety inspections completed in time versus percentage of safety inspections conducted in a qualitative manner).
- Differentiate safety indicators based on relevant differences regarding positions or departments (e.g., percentage of received safety training for employees, contractors, top management, etc.).
- Answer categories should include enough gradations (e.g., only including a yes/no possibility for an answer is often too one-sided).

These recommendations are taken into account during the next step of developing a methodology for composing company-specific indicators, as are the recommendations that have been formulated based on the review of existing tools (see step A Figure 3), such as using terms that are tailored to the common vocabulary in the organisation to optimise the familiarisation of the safety indicators for the respondents.

An overview of the final observable sub-domains and their definitions can be found in Table 9.

#### 5.3.2. Development Methodology for Composing Company-Specific Indicators

**Figure 5:**
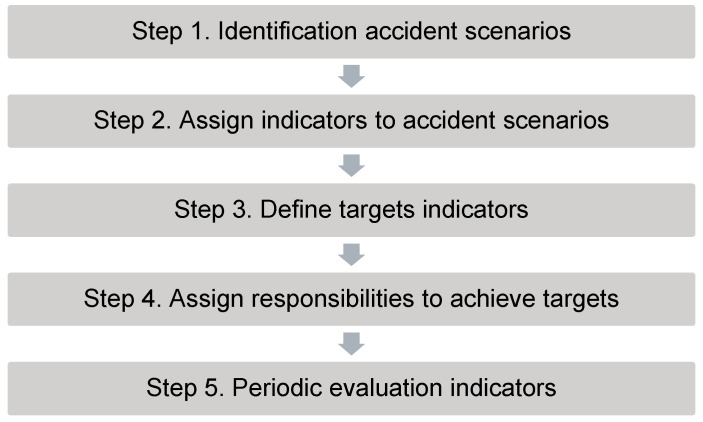
Different steps in the methodology for composing company-specific indicators.

The methodology that enables organisations to compose a set of company-specific indicators in order to operationalise the observable sub-domains of safety culture has been elaborated in van Nunen et al. [127,128]. The methodology consists of five different steps (see Figure 5). In this part, a brief summary of this five-step approach is given.

Step 1. Identification accident scenarios

The first step is the identification of possible accident scenarios within the company. In the articles of van Nunen et al. [127,128], the use of the bow-tie method is explained in-depth as a suitable method to make an inventory of possible accident scenarios, taking into account the specific context of an organisation. When using this bow-tie approach, a comprehensive and detailed overview can be composed of possible causes and consequences of potential incidents within a company. Bow-ties also include the influence of safety measures, i.e., safety barriers and management delivery systems, on the evolution of accident scenarios. The bow-tie method also makes a clear distinction between preventing and mitigating safety barriers and management delivery systems, where the preventing safety barriers are situated before a central event, and where the mitigating safety barriers are situated after the occurrence of a central event.

Step 2. Assign indicators to accident scenarios

Once bow-ties are composed, they form an excellent point of departure to assign indicators to the safety barriers and to the management delivery systems in order to control (i.e., prevent or mitigate) the accident scenarios. When safety indicators are composed by using possible accident scenarios as a point of departure, it is ensured that the specific needs and context of a company are taken into account.

When composing safety indicators, it should be monitored that both reactive indicators and proactive indicators are included. Reactive or lagging indicators focus on what goes wrong and often use accidents as a basis, such as the number of accidents and severity rate of accidents. Proactive or leading indicators precede an unwanted event (or central event) and focus on what goes well, such as the quality of training and timely completed actions of safety rounds.

Another point of attention is the sequence of follow-up of safety indicators. This means that indicators looking at the quality of safety measures take precedence over the quantity of safety measures. Indicators assigned to a management delivery system such as ‘training of operators’ could be, for instance, about the coverage ratio of the training (percentage of operators receiving safety training), and about the content of the training (the extent to which the safety training is tailored to the needs of the company). The sequence in follow-up does not imply that the indicator targeting the coverage ratio of the training is less important, but it means that this indicator is not useful if the indicator targeting the content of the training is not met. It could be that 100% of the operators received safety training, but if the quality of this training is substandard, a full coverage ratio is insignificant.

It is recommended that safety indicators are defined according to the SMART framework, where SMART is an acronym for Specificity, Measurability, Achievability, Relevancy, and Timeliness [129].

Step 3. Define targets indicators

Once safety indicators are composed, the next step is to define targets for each indicator. This is a subjective allocation of what the organisation finds desirable and acceptable as a result. For example, a target for the training of operators could be that 100% of all operators receive qualitative safety training. It is important that targets are realistic. This could imply a gradual move toward the target. For instance, in year x, 80% of all operators need to be trained, and in year y, 100% must be trained.

Step 4. Assign responsibilities to achieve targets

The next step is to assign responsibilities to achieve the targets being set. For example, who takes care of developing training that is tailored to the needs of the company? Or who takes care of the registration of the operators for the training? These responsibilities should be divided among employees and supervisors and also among the (higher) management.

Responsibilities must also be defined if targets are not met. In addition, again, when setting targets, these responsibilities must also be realistic. The required efforts must be achievable, and those responsible must have sufficient knowledge, capacity, resources, and authority to perform their tasks.

Step 5. Periodic evaluation indicators

The last step is the periodic evaluation of safety indicators. Depending on the specificity of the indicator, the indicator must be evaluated, for example, once a year or once a month. The need for this periodic evaluation emphasizes the importance of continuous follow-up when assessing the safety culture of an organisation, as visualised in Figure 4.

To evaluate a safety indicator, information or data need to be collected. This data collection can be achieved by means of (a) document analysis where information is gathered from internal documentation such as an organisation’s policy statements, website, intranet, etc. [19], (b) analysis of registered data such as incident registrations, and/or (c) observations in the organisation, such as observations of the working environment and behaviour of employees.

Unlike the questionnaire to measure the non-observable safety culture domains, which preferably should be completed by everyone in the organisation, the safety indicators only have to be evaluated by one person. After all, safety indicators represent objective information (there is only one correct answer), while the questionnaire is about subjective information (there is no right or wrong answer). The company itself decides which person (or department) is best suited to provide the information to evaluate a specific safety indicator. This person needs to have access to reliable and accurate data within the company, in order to be able to complete the objective safety indicators as correct as possible. It could be possible that the needed data to evaluate a safety indicator is not available, for instance, because no records are being kept within the organisation. It is then recommended to register this information in the future. 

**Table 9:** Observable domains of safety culture and their sub-domains.

Technological Domain (Characteristics Working Environment)		
Sub-Domain	Definition of Sub-Domain	Relevant References (Step C Figure 3)
Physical working environment	The characteristics of the working environment, i.e., the job demands and the level of risk at the workplace, and their corresponding safety measures: - Risk analysis: Up-to-date? Employees involved? … - Safety measures: Consideration of both preventive measures (e.g., adaptation production process) as mitigating measures (e.g., emergency response plan)? It also includes the use of existing technology, processes, design, materials, equipment,…: - Purchase: Is safety taken into account when purchasing? Are alternatives compared as regards safety? … - Maintenance: Is maintenance done on a regular basis and in due time? …	[19,21,37,42,45,69,130,131,132,133]
**Organisational domain (safety management)**		
**Sub-domain**	**Definition of sub-domain**	**Relevant references (step C Figure 3)**
Safety policies and goals	Is there a safety vision and mission and corresponding safety strategies to reach this vision and mission, and to what extent is this included in the company policies? Have safety objectives been set (for the organisation as a whole/for departments separately) and are these safety objectives followed up?	[19,131,134,135,136]
Safety performance	The extent to which safety objectives are met or not. For instance: - A minimal or decreasing number of incidents or accidents (occupational or process related), occupational diseases,… - A maximal number of (new) employees receiving a safety education and training	[137,138,139]
Resources for safety	Are sufficient resources (budget, time, people) available to reach safety objectives such as adequate safety (re)training, safety communication,…	[19,138,140]
Safety communication and transparency	Is relevant safety information (e.g., the safety performance of the company as a whole/for departments separately, the relevant contact persons for safety related issues,…) communicated in a clear, accessible way (tailored to the target group) on a regular basis? How well is ‘safety’ represented as a topic within commonly used communication channels (e.g., intranet, newsletter, e-mail,…)? Is safety a topic during one-to-one conversations, such as during an evaluation interview?	[141,142,143,144]
Safety inspection	Are safety rounds performed on a regular basis? Are action points that are being formulated based on these safety rounds completed within the stipulated time frame? Is (top) management also involved in these safety rounds?	[21,145,146,147]
Safety registration and follow-up	Registration/reporting of unsafe situations, incidents, near-misses, accidents.Follow-up of these registrations/reports such as: - Is an (incident/accident) analysis performed? - Are measures taken to prevent a similar event in the future? - Is the workforce involved in formulating these safety measures? - Is feedback given afterwards to the person who made the registration (also when no measures are being implemented)?	[19,109,148,149]
Safety procedures and instructions	Is safety an integral part of work procedures and work instructions, and is it included in a clear and accessible way (tailored to the target group)? Are procedures and instructions revised (are they still up-to-date?) on a regular basis and adjusted if needed? Is the workforce and/or safety department involved when procedures and instructions are composed or revised?	[19,21,150,151,152]
Safety and contractors	Initiatives to ensure a safe environment when working with contractors, such as: - Including safety as a selection criterion for contractor companies, - Agreements regarding safety obligations between the contracting company and contractor company, monitoring these agreements and taking action when there are non-compliances, - Provision of clear and accessible safety information and/or safety training for contractors, - Regular meeting with the contractor company including safety as a topic, - Giving contractors the opportunity to report unsafe situations.	[89,153,154,155,156]
**Human domain (safe and unsafe behaviour)**		
**Sub-domain**	**Definition of sub-domain**	**Relevant references (step C Figure 3)**
Safety compliance	Adhering to safety procedures and carrying out work in a safe manner; i.e., posing safe or unsafe behaviour based on the working activities within the organisation and relevant accident scenarios, such as correctly wearing personal protective equipment (PPE), applying correct lifting techniques, adherence to the speed limit on-site, conducting last-minute risk analysis (LMRA) if needed, adherence to Lock-Out Tag-Out (LOTO) procedures if needed, stacking loads correctly when driving a forklift, order and tidiness, …	[21,44,131,157,158,159,160]
Safety participation	Safety behaviour that goes beyond the formally established role of the workforce (as explained in the sub-domain ‘safety compliance’) such as reporting safety improvement ideas, spontaneously helping co-workers as regards to safety, promoting the safety program within the workplace,…	[19,21,44,71,81,157,161]

**Table 10:** Example of sub-domain results per position (Score from 1 to 5).

	Employee (n = 271)	Supervisor (n = 104)	Management (n = 48)	Safety Department (n = 6)	Contractor (n = 15)	Total Score (n = 444)
Non-observable organisational domain						
Dealing with accidents	3.8	3.8	4.1	3.4	*	3.8
Supervisor commitment and leadership	3.8	4.0	4.1	3.3	*	3.9
Dealing with near-misses	3.1	3.1	3.0	3.2	*	3.1
Victim blaming	3.1	3.3	3.6	3.3	*	3.2
Management commitment	4.0	4.1	4.1	3.7	*	4.0
Priority for safety	4.1	4.1	4.0	3.7	4.2	4.1
Employee commitment	3.3	3.4	3.4	2.8	*	3.3
Involving employees	3.9	3.9	4.0	4.2	3.9	3.9
Safety department commitment	4.1	3.9	4.1	*	*	4.0
Impact safety department	*	3.8	3.8	3.7	*	3.8
Commitment towards external parties	3.8	3.8	3.9	3.7	4.4	3.8
Commitment from external parties	3.4	4.5	3.4	3.2	*	3.4
Supporting environment: time and people	3.6	2.7	3.8	3.2	3.7	3.6
Supporting environment: safety education	4.1	4.0	3.9	4.2	*	4.1
Supporting environment: addressing inconveniences	3.0	3.1	3.4	3.7	*	3.1
Supporting environment: safety rules	3.6	3.6	3.5	4.0	3.8	3.6
Non-observable human domain						
Personal priorities	4.4	4.4	4.6	*	4.3	4.4
Safety responsibilities	4.5	4.6	4.7	4.3	4.8	4.6
Intention for proactive safety behaviour	3.9	3.9	4.1	*	4.6	4.0
Overall safety knowledge and competence	3.9	3.9	3.1	*	4.5	3.8
Knowledge and competence during safety problems	4.2	4.1	4.0	*	4.5	4.2
Trust in the organisation	4.0	3.9	4.1	3.7	4.0	4.0
Intention to behave safely/unsafely	4.1	4.2	4.0	*	4.3	4.2
Intention to behave safely/unsafely under pressure	4.3	4.3	*	*	3.9	4.3

**Figure 6:**
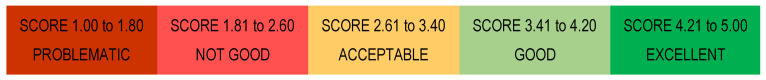
Interpretation of the questionnaire results.

For an interpretation of the colours, see Figure 6. * No questions from this sub-domain were questioned within this position.

### 5.4. Results of the Quantitative Scan

As explained in the previous parts, a set of questions has been developed to assess the non-observable safety culture domains, and a method to compose a set of company-specific safety indicators has been developed to assess the observable safety culture domains. The following part explains how the completed questionnaires and safety indicators are analysed and how the results can be interpreted.

#### 5.4.1. Results and Interpretation of the Questionnaires

When completing the questionnaires to assess the non-observable safety culture domains, respondents have answering possibilities in the form of a five-point Likert scale ranging from (1) disagree completely to (5) agree completely. For questions formulated in a negative way, the score is reversed. When a question is answered with the ‘not applicable’ option, the answer is omitted in further analysis.

After the questionnaires have been completed, the average score on each separate question is calculated. Subsequently, the score on each sub-domain is determined by calculating the average score of all questions belonging to a specific sub-domain. In addition, as the last step, the average of all the scores on the sub-domains represents the score on the associated safety culture domain. All scores—on the separate questions, on the sub-domains, and on the safety culture domains—range from 1 to 5. The scores are translated into five categories ranging from problematic to excellent (see Figure 6).

When analysing the questionnaires, the results of the safety culture domains and sub-domains can also be disaggregated according to the specific characteristics of the respondents. As the content of the questionnaires differs according to the position of the respondents (see also Section 5.2.1), results will evidently be differentiated according to the different positions. Table 10 shows an example of the results per safety culture sub-domain for the different positions of the respondents. 

Besides the position of respondents, other levels of aggregation are also possible. As stated by Zohar [162], conditions determining the appropriate level of analysis require within-group homogeneity and between-groups variance, such as the different departments within an organisation [21,163]. Differences in results between departments can provide an insight into the different sub-cultures of an organisation, which can, according to several authors (e.g., [35,164,165], coexist within an individual organisation.

Other levels of aggregation are possible, under the precondition that each aggregation should have reasons for being viewed as a group [21]. Possibilities are background characteristics of respondents such as their seniority or their age [166], their gender, the highest level of education achieved, or working time present on site.

When disaggregating results, it should be acknowledged that the number of respondents per aggregation level decreases, which could compromise the anonymity of respondents. In order to increase the response rate of a questionnaire and to receive reliable answers, guarantying anonymity of respondents is, however, considered a key aspect (as well as other aspects such as making the completion of the questionnaire voluntary, and emphasising that there are no right or wrong answers). The minimum number of 30 respondents is often taken as a rule of thumb to report results [167]. When groups are smaller than this minimum number of 30 respondents, groups can be merged to report results, for instance, by merging departments with similar activities, or by merging consecutive age groups.

In practice, it will, however, not always be possible to reach the minimum number of 30 respondents per aggregation level because of a low response rate or questioning a small organisation. Reporting the results even though the number of respondents is less than 30 is possible, under the precondition that this is being adequately framed. Adopting a ‘no-blame policy’ is needed, whereby it is assured that the purpose of the assessment is to learn and to improve, and not to punish and point fingers. If respondents do not have to worry about possible negative consequences due to lack of anonymity when reporting the results, such as a negative reaction from a colleague, a verbal warning from a supervisor, or even an entry in the respondent’s personal file, then it is possible to work with a small(er) number of respondents. Good communication with the entire organisation is key in this regard (see also Section 5.7).

#### 5.4.2. Results and Interpretation of the Safety Indicators

**Figure 7:**
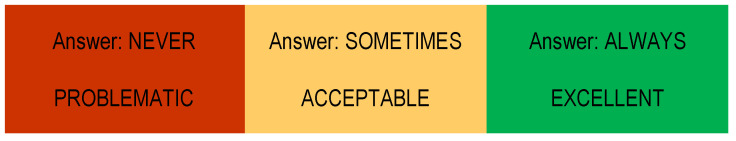
Example of the interpretation of a safety indicator result.

The answering possibilities for the safety indicators vary, and include, amongst others, a percentage, a number, a dichotomous scale such as yes/no, or an ordinal scale such as never/sometimes/always. The conversion of the answer options of the various safety indicators to a score is always different and is determined per indicator. When company-specific indicators are composed (see Section 5.3.2), the third step is to define targets for each safety indicator, which includes the categorisation of the scores. Figure 7 shows a possible interpretation of a safety indicator with the answering possibilities never/sometimes/always.

#### 5.4.3. Comparison Results between Observable Safety Culture Domains and Non-Observable Safety Culture Domains

**Figure 8:**
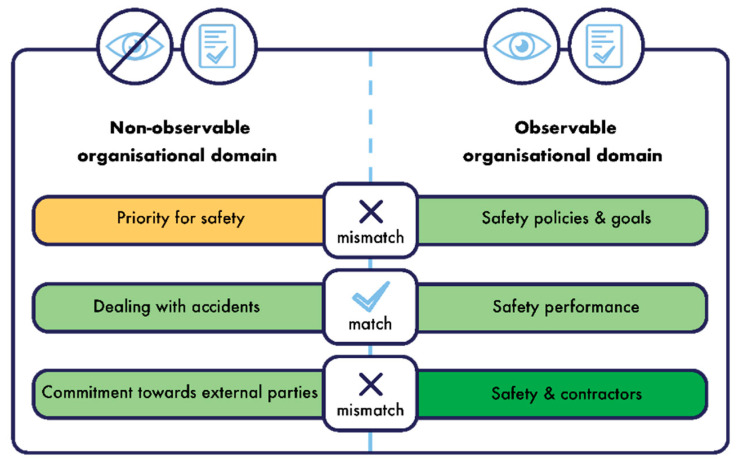
Example of a comparison between the results of the observable sub-domains and non-observable sub-domains.

As a result of the quantitative scan, a score and corresponding categorisation ranging from problematic to excellent are calculated for all safety culture domains and sub-domains. Another important and insightful result is the comparison of the results within the observable safety culture domains and the non-observable safety culture domains. This comparison exhibits to what extent there is a match or a mismatch between the results of the observable (sub-)domains (objective safety-related aspects) and the results of the non-observable (sub-)domains (subjective safety-related aspects). Figure 8 shows an example of such a comparison. 

For instance, the safety indicators (objective, observable) could show that many resources are spent on safety communication. On the other hand, the questionnaire (subjective, non-observable) could reveal that employees have the feeling that they are not adequately informed about the safety issues affecting them. Or it may objectively appear that a lot of safety training is followed by employees, but it may also subjectively appear that employees feel that the offered safety training is not tailored to the specific needs of the organisation.

#### 5.4.4. Benchmarking

Benchmarking is a business practice that aims at comparing performance and consequently improving this performance [168]. By comparing performances, benchmarking broadens an organisation’s experience base, as it provides insights into the experiences of better-performing parties, as well as insights into those things that do not work so well [169]. In this sense, benchmarking supports a learning organisation [169].

Different types of benchmarking exist, such as ‘competitive’ or external benchmarking, and internal benchmarking [170]. External benchmarking compares performances—in this case, safety culture performances—across different organisations. It enables individual organisations to evaluate their safety culture position relative to other organisations [170]. It is important that this type of benchmarking is not competitive-oriented with the underlying goal to be better than other organisations, but rather that it is learning-oriented, where the benchmarking position functions as an initiation for continuous safety culture improvement. A prerequisite for external benchmarking is that a large pool of organisations participates in the safety culture assessment. 

Internal benchmarking compares performances within the same organization [170]. This includes a comparison between different business units or between different locations of the organisation (if any) and a comparison between different measurements within the same organisation. The latter implies a longitudinal comparison, and is a key aspect as regards the follow-up of safety culture assessment (see Figure 4). Comparing different safety culture measurements in time enables the determination of a change, i.e., a progress, status quo, or a deterioration regarding the organisational safety culture.

### 5.5. In-Depth Analysis

In the ISCA-approach, the first step in diagnosing the safety culture of an organisation is conducting the questionnaires and completing the safety indicators. The next step is also part of diagnosing the safety culture, and is a more in-depth analysis (see Figure 4). Where the questionnaires and safety indicators focus on a quantitative approach, the in-depth analysis uses a qualitative methodology. During this in-depth analysis, the results deriving from the questionnaires and safety indicators are further explored by means of interviews and/or focus groups with all layers of the organisation. This qualitative approach is needed to provide a deeper insight into complex contexts, and to learn more about the specific underlying causes and nuances of the results. The sub-domains needing improvement should be further investigated during this in-depth analysis, but also the sub-domains with excellent scores should be further looked into, as these insights can similarly be used during the next step of the safety culture assessment, namely the formulation of safety culture improvement strategies. Specifically for the questionnaires, significant differences in the disaggregated results according to specific characteristics of the respondents should be looked into. If there are significant differences between the results of, for instance, positions, or departments, underlying causes must be mapped, so that improvement strategies can be tailored to these differences. The in-depth analysis should also pay attention to the differences between the observable sub-domains and the non-observable sub-domains (see Figure 8 for an example) and look into possible causes of discrepancies or mismatches.

### 5.6. Formulation of Recommendations and Implementation of Improvement Strategies

**Figure 9:**
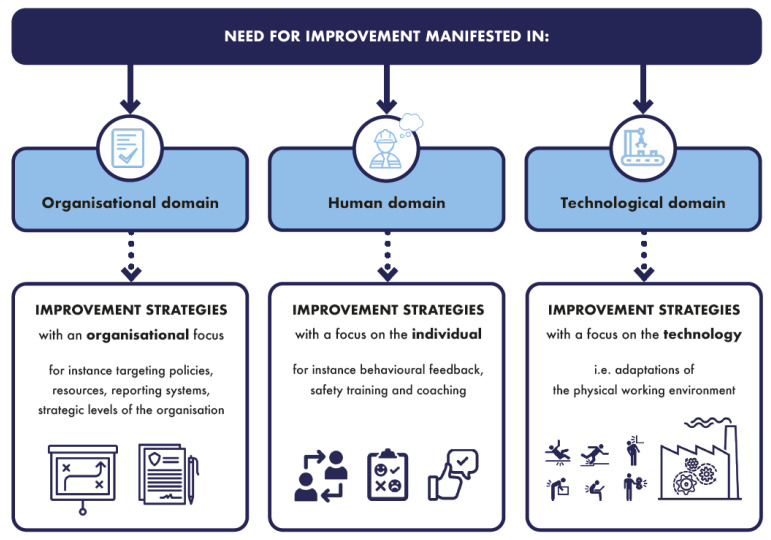
Focus of the safety culture improvement strategies.

After the diagnosis of the organisational safety culture, the next steps in the ISCA-approach can be performed, namely the formulation of recommendations and the implementation of improvement strategies (see Figure 4). The diagnosis of the safety culture, so both the quantitative results derived from the questionnaires and safety indicators, and the qualitative results derived from the in-depth analysis, form the basis for the formulation of recommendations. Improvement strategies should be developed in such a way that they address/improve the safety culture (sub)domains with less good scores, and that they maintain the statuses of the (sub)domains with currently good scores. When formulating and implementing improvement strategies, some key aspects should be taken into consideration:

- It is needed to keep a short time period between the safety culture diagnosis on the one hand, and the formulation of recommendations and implementation of improvement strategies on the other hand. As mentioned by Davies et al. [58], some visible results need to be achieved as soon as possible after a safety culture measurement. When formulating recommendations, priorities can be set, and differentiations can be made between ‘quick wins’ that are feasible to implement in the short term, and structural changes focussing on the longer term.
- The involvement of the entire organisation is key when diagnosing the safety culture, and when formulating recommendations. When all layers of the organisation are involved in setting up improvement strategies, support will be raised increasingly, and a kind of guarantee is built that recommendations are realistic. During the in-depth analysis, ideas for improvement strategies can already be a topic of discussion.
- Safety culture results differ according to, for instance, positions or departments. Improvement strategies should be tailored to these differences in order to increase their success ratio.
- When formulating improvement strategies, it is important to keep in mind to which domain the aspects needing improvement belong. If the need for improvement is manifested in the organisational domain, the recommendations need to have an organisational focus. On the other hand, if the need for improvement is manifested in the human domain or in the technological domain, the recommendations need to focus on the individual or on the technology, respectively (see Figure 9). For instance, a result of the questionnaire could indicate that the sub-domain ‘safety rules’ scores below average. The sub-domain ‘safety rules’ belongs to the organisational domain and includes the extent to which the safety rules are clear and non-redundant. Consequently, improvement strategies aiming at improving this organisational sub-domain must have an organisational focus, for instance, how can the clarity of safety rules be improved for the entire organisation.

### 5.7. Points of Attention When Assessing Safety Culture

During the entire process of the safety culture assessment, communication is crucial. Before starting the safety culture measurement (the first step in Figure 4), all employees should be informed about the purpose. It should be clarified why the organisation is conducting a safety culture measurement, why participation is important, and how the results will be used. After the safety culture measurement, the results should be communicated, preferably as soon as possible, after the measurement [171]. Further, the next steps that will be taken based on the results of the measurement should be communicated.

Another important point when assessing safety culture is acknowledging that it is a never-ending process. When improvement strategies are implemented, also some form of monitoring needs to be in place to check the progress and effectiveness [58]. As seen in the ISCA-approach visualised in Figure 4, a continuous trajectory needs to be followed. In practice, this continuous trajectory is often not followed. For example, there are companies that conduct a measurement, but do not implement any changes afterwards [13]. As a result, an organisation can go backward instead of forward. It is, therefore, necessary that companies are engaged to work on their safety culture with a long-term view in mind.

This long-term engagement implies that after improvement strategies are implemented, a follow-up measurement needs to be performed to check whether the implemented recommendations have indeed achieved their goal; however, a follow-up measurement should not be undertaken until the improvement strategies put in place have been allowed sufficient time to generate results [58]. The time interval between two measurements needs to be long enough so that improvements manifest themselves within the organisation to such an extent that it would lead to significantly different outcomes of the measurement [58].

Furthermore, full support and dedication from top management are crucial when a safety culture assessment trajectory is started [19]. This management commitment can be expressed by, for instance, assuring to adequately act upon the findings of a safety culture measurement. Top management also has an important role in involving the entire workforce throughout the process [58]. The involvement of the entire organisation is key when diagnosing the safety culture (to obtain the most accurate picture possible), and it is also crucial during the next steps of formulating recommendations and implementing improvement strategies. By, for instance, propagating a ‘no-blame policy’, where it is guaranteed that safety culture measurement and assessment have the goal of learning and improvement, and not to punish, top management can motivate the entire workforce to take up a role.

Related to this management commitment is the ‘willingness to change’ that needs to be present. Of course, if an organisation decides to start a safety culture improvement programme, this already implies a certain willingness to change; however, the initiative to start such a programme should not be originated from the moral obligation to corporate social responsibility and involvement, but should be founded into the genuine desire to bring about a positive change. The chance of success will be lower within organisations with a ‘we have to’ mentality, compared to organisations with a ‘we want to’ mentality.

When measuring safety culture, the involvement of the entire organisation is needed to obtain the most complete picture possible; therefore, for the quantitative scan of the ISCA-approach, a web application is developed to collect the needed data in an accessible way; however, to also include workers not having access to a computer, the data for the quantitative scan can also be collected on paper, for a part of the organisation or the entire organisation.

## 6. Discussion

**Figure 10:**
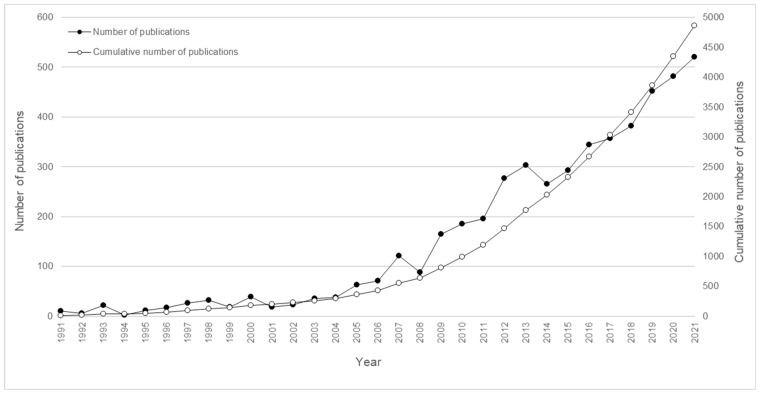
Number of safety culture publications and cumulative number of safety culture publications by year (1990–2021) (Adapted from [2]).

The complexity and multifacetedness surrounding ‘safety culture’ results in a vast amount of research aiming at exploring, understanding, influencing, and managing safety culture in organisations. As elaborated in the article of van Nunen et al. [2], 1789 publications related to safety culture were published between 1900 and 2015. An updated search in Web of Science, including articles on safety culture published until 2021, shows that this number has exponentially increased to 4866 (see Figure 10), representing an increase of 272%. Due to this large number of publications, performing a literature search on safety culture, and other related concepts, such as safety climate and safety performance, inevitably implies falling down the metaphorical rabbit hole, where this metaphor refers to an extremely engrossing and time-consuming topic. Paradoxically, although the rabbit hole of safety culture leads to an overwhelming variety of metaphors, models, and theories, safety culture research simultaneously has drawn on reductionism. Reductionism is the practice of simplifying the description of a complex phenomenon in order to better grasp it [172]. The concept of safety culture has been characterised by reductionism: many theoretical and practical approaches to safety culture are reduced to, for instance, a simplified metaphor or a quick checklist or questionnaire, or by equating safety culture to safety performance or behaviour.

In this article, the approach of reductionism has been discarded by developing an integrative and comprehensive model to assess safety culture in organisations. Technological factors (such as technology, processes, and materials), organisational or contextual factors (such as policies, resources, and supporting environment), and human factors (such as knowledge, intentions, compliance); all are taken into account as interacting and interrelating elements in the ISCA-model. In this sense, the totality of factors that define a safety culture of an organisation are considered instead of the general tendency to focus on one particular set of factors [37,173]. The comprehensiveness of ISCA lies in the inclusion of technological factors, organisational factors, and human factors, and in considering both observable or objective safety-related aspects in an organisation, and non-observable or subjective safety-related aspects. In addition, the comparison between these objective and subjective safety-related aspects has a pivotal role in the ISCA-approach. What looks safe on paper (for instance, all employees received safety training) may not correspond to what people feel, think, or experience in practice (for instance, employees feel that they lack the training to perform their tasks in a safe way).

## 7. Conclusions

### 7.1. The Development of ISCA

To develop ISCA, a multi-phased method was used. A review was performed of existing practical tools to measure and improve organisational safety culture, in order to identify the strengths and limitations of these existing tools. This led to the overall ISCA-approach, defining that safety culture must be assessed in an integrative way by using a variety of research methods involving the entire organisation, and by taking into account the specific context of the organisation (as there is no such safety culture measurement as a ‘one size fits all’).

The content of ISCA was initially developed based on a literature review to ensure that all existing theoretical concepts for assessing safety culture were covered. After the literature review, the non-observable part of ISCA (mapping all subjective safety-related aspects) and the observable part (mapping all objective safety-related aspects) were further operationalised. 

For the non-observable part of ISCA, questionnaires were developed to measure the perceptual domain and psychological domain of safety culture. For each sub-domain, questions were formulated based on the literature review. Content validity and face validity were tested extensively by means of several focus groups with 84 experts, and by pre-testing the questionnaire in two different companies with a total of 291 respondents. This led to a draft version of the questionnaire, of which different versions were available, depending on the position of the respondent (employee, supervisor, manager, employee at the safety department, or external). This draft version of the questionnaire was tested on construct validity (both exploratory and confirmatory) with a sample of 444 workers at a Belgian chemical company. This construct validity was only tested for questions applicable to (at least) the employees. This is because—due to the hierarchical structure of companies—the total numbers of supervisors, managers, and staff members of the safety department, are too low to perform the analyses. The EFA phase for the perceptual domain resulted in a ten-factor solution from 32 items, which retained 74.4% of the variance. All items kept for the solution showed a minimum factor loading of 0.400 on their respective factors, and no cross-loadings were admitted. The solution for the psychological domain retained five factors from 16 items and reflected 69.3% of the total variance. A confirmatory factor analysis was used to further purify the factors. The final solution for the perceptual domain resulted in 27 items, which formed ten factors. The final solution for the psychological domain had 15 items forming five factors and successfully passed convergent and discriminant validity.

For the observable part of ISCA, the focus groups with 84 experts also considered the content validity of the sub-domains as formulated based on the literature review. Correspondingly, to measure these sub-domains, an approach to composing company-specific safety indicators has been developed. So, for the observable part of ISCA, the sub-domains themselves are fixed, but the operationalisation of these sub-domains is not. The approach to composing company-specific safety indicators consists of different steps, of which the first step is identifying relevant accident scenarios within the company, including safety barriers and management delivery systems influencing the evolution of these accident scenarios. Accident scenarios can cover the entire spectrum of probability (from high to low) and consequences (from absent to severe), as long as they are relevant to the company. The second step in the approach is to assign indicators to the relevant accident scenarios, safety barriers, and management delivery systems. In doing so, it is important to consider the quantitative outcomes of safety indicators, and also the qualitative outcomes hereof. For instance, an indicator could measure how often safety is an item on the agenda of important management meetings (quantitative outcome), but it should also be looked into if this safety topic is covered thoroughly or seriously during these meetings (qualitative outcome). Other steps when composing safety indicators are defining achievable and realistic targets, and assigning responsibilities to achieve these targets. As the developed approach to compose a set of company-specific safety indicators uses the relevant accident scenarios within a certain company as a starting point, it is ensured that the objective safety culture measures are tailored to the specific needs and context of the company.

The quantitative safety culture scan using questionnaires and safety indicators is followed by an in-depth qualitative analysis using interviews and/or focus groups to explore the specific underlying causes and nuances of the results. This in-depth analysis should cover the underperforming (sub-)domains of safety culture as identified during the quantitative scan. Further, the currently good or excellent performing (sub-)domains should be the topic of discussion during the in-depth analysis, as this can gain valuable insights (what can we learn from this?).

Following the quantitative scan and in-depth qualitative analysis, the next step in the ISCA-approach is formulating and implementing improvement strategies. In addition, in this step, as is needed for measuring and validating results, the involvement of the entire company and all layers of the organisation is important.

Key in assessing safety culture is the continuity of the approach. Assessing safety culture is a never-ending process where implemented improvement strategies should be followed up, and it should be monitored if (sub-)domains needing improvement indeed progress over time, and if good or excellent performing (sub-)domains remain stable and do not deteriorate over time. 

### 7.2. Strengths and Limitations of ISCA

One of the strengths of the ISCA-approach is that throughout the entire process of developing ISCA, the working field was closely involved, both content-wise as regards what constitutes the safety culture of an organisation, and as regards the used approach of assessing safety culture. This close involvement of the working field, combined with the comprehensive literature review that was performed, leads to an approach founded in science but practically usable in the field. 

Another strength is the inclusion of compared results between the observable safety culture domains and the non-observable safety culture domains. A mismatch between the objective safety-related aspects and the subjective safety-related aspects exposes specific points of attention and improvement for the organisation under investigation. 

Furthermore, dividing the ISCA framework into domains and sub-domains has the advantage of aligning improvement strategies with specific (sub-)domains needing improvement. Points of improvement can be addressed and targeted in a tailored way which prevents that, for instance, another safety training is raised as an improvement strategy, while the possibility for improvement actually lies somewhere else.

Notwithstanding the aforementioned strengths and advantages, the ISCA-approach has some limitations that should be considered. Firstly, the comprehensiveness of ISCA makes scientific validation difficult. Regarding the questionnaire part, factor analysis could not be performed for questions only applicable to the supervising, management, and safety positions due to the lower number of respondents belonging to these positions. This implies that not all sub-domains in the ISCA framework are validated by confirmatory factor analysis (see sub-domains without * in Figure 2). Further testing of the questionnaire is therefore recommended by administering the questionnaire in different organisations to achieve a sufficient number of supervisors, managers, and employees of the safety department to conduct factor analysis.

The ISCA model and approach are developed in such a way that they can be used in all types of organisations, regardless of the sector or company size. However, the model and approach can seem overwhelming, definitely for SMEs (small and medium-sized enterprises) where several parts of the model are not applicable, such as using the division in different positions or working with contractors.

Another shortcoming is that the organisations participating in the development of ISCA are organisations that have already reached a certain maturity regarding safety culture. In the development, organisations that are disadvantaged regarding safety culture are not included, as these organisations are difficult to reach and to motivate for participation. It is, therefore, difficult to estimate to which extent the approach is applicable to organisations where significant improvement regarding safety culture is still needed. 

Finally, the cross-sectional design should be mentioned as a limitation. Further research with a longitudinal setup is needed in order to draw conclusions about the effect of the assessment on the improvement of the organisations’ safety culture over time.

## Figures and Tables

**Table 1:** Descriptive characteristics of the study samples.

	Company A (n = 144) Number (%)	Company B (n = 147) Number (%)	Company C (n = 444) Number (%)
**Position**			
Employee	47 (32.6)	96 (65.3)	271 (61.0)
Supervisor	10 (6.9)	23 (15.7)	104 (23.4)
Manager	9 (6.3)	4 (2.7)	48 (10.8)
Staff member safety department	5 (3.5)	4 (2.7)	6 (1.4)
External	73 (50.7)	20 (13.6)	15 (3.4)
**Age (years)**			
18–35	34 (23.6)	18 (12.6)	68 (15.3)
36–45	30 (20.8)	43 (30.1)	111 (25.0)
46–55	54 (37.5)	62 (43.3)	174 (39.2)
56 and above	26 (18.1)	20 (14.0)	91 (20.5)
**Highest level of education**			
No degree or primary education	15 (10.5)	11 (7.8)	30 (6.8)
Secondary education	72 (50.3)	75 (53.2)	140 (31.8)
Bachelor’s degree	21 (16.1)	47 (33.3)	218 (49.6)
Master’s degree	33 (23.1)	8 (5.7)	52 (11.8)
**Seniority (years)**			
<1	14 (9.7)	9 (6.3)	39 (8.8)
1–5	31 (21.5)	16 (11.2)	85 (19.2)
6–10	24 (16.7)	25 (17.5)	62 (14.0)
11–15	17 (11.8)	20 (14.0)	29 (6.5)
16–20	18 (12.5)	47 (32.9)	58 (13.1)
≥21	40 (27.8)	26 (18.1)	170 (38.4)

**Table 2:** Exploratory factor analysis (n = 444)—items related to the perceptual domain.

Items	Factor									
	1	2	3	4	5	6	7	8	9	10
Id.1312	**0.704**	0.114	0.127	−0.015	0.064	0.064	0.116	0.123	0.023	0.081
Id.1314	**0.606**	0.287	0.140	0.061	0.170	0.107	0.139	0.054	0.212	0.134
Id.1315	**0.570**	0.053	0.103	0.096	0.364	0.084	0.140	0.055	0.169	0.081
Id.1313	**0.548**	0.262	0.242	0.219	0.173	0.068	0.099	0.008	0.116	0.191
Id.1298	**0.509**	0.055	0.233	0.305	0.192	0.086	0.089	0.081	0.093	0.057
Id.1297	**0.490**	0.038	0.211	0.288	0.163	0.049	0.177	0.130	0.060	0.026
Id.1319	**0.419**	0.080	0.200	0.098	0.360	0.146	0.103	0.034	0.161	0.195
Id.1307	**0.401**	0.071	0.132	0.074	0.358	−0.010	0.200	0.200	0.126	−0.066
Id.1309	0.135	**0.871**	0.160	0.102	0.079	0.093	0.092	0.003	0.096	0.162
Id.1308	0.136	**0.805**	0.126	0.170	0.114	−0.026	0.123	0.034	0.049	0.160
Id.1310	0.157	**0.560**	0.103	0.050	0.069	0.162	0.118	−0.054	0.175	0.121
Id.1340	0.204	0.089	**0.679**	0.037	0.153	0.007	0.002	0.093	0.097	0.122
Id.1339	0.272	0.071	**0.597**	0.224	0.021	0.027	0.194	0.181	0.042	0.078
Id.1341	0.120	0.160	**0.573**	0.072	0.115	0.142	0.086	0.128	0.105	0.154
Id.1342	0.134	0.172	**0.502**	0.190	−0.005	0.078	0.137	0.002	0.230	0.091
Id.1300	0.125	0.160	0.142	**0.832**	0.136	0.017	0.146	0.078	0.096	0.126
Id.1301	0.213	0.146	0.183	**0.806**	0.132	0.021	0.147	0.122	0.083	0.083
Id.1316	0.315	0.088	0.058	0.118	**0.689**	0.002	0.181	0.086	0.098	0.109
Id.1317	0.262	0.161	0.144	0.181	**0.651**	0.000	0.157	0.277	0.103	0.046
Id.1318	0.335	0.221	0.109	0.113	**0.416**	0.080	0.181	0.073	0.193	0.314
Id.1302	0.114	0.086	0.084	0.052	0.024	**0.943**	0.044	−0.015	0.083	−0.005
Id.1303	0.110	0.085	0.082	−0.006	0.023	**0.911**	0.016	−0.049	0.065	0.003
Id.1332	0.186	0.177	0.103	0.157	0.222	0.003	**0.739**	0.132	0.117	0.198
Id.1331	0.212	0.137	0.085	0.165	0.134	0.040	**0.597**	0.224	0.035	0.065
Id.1330	0.249	0.135	0.279	0.095	0.163	0.073	**0.508**	0.181	0.145	0.117
Id.1320	0.124	0.021	0.138	0.102	0.099	−0.062	0.186	**0.949**	0.013	0.062
Id.1321	0.168	−0.081	0.186	0.105	0.234	−0.026	0.205	**0.608**	0.053	0.133
Id.1328	0.162	0.150	0.175	0.108	0.126	0.055	0.081	0.035	**0.931**	0.074
Id.1327	0.198	0.148	0.184	0.074	0.152	0.121	0.108	0.040	**0.652**	0.111
Id.1326	0.089	0.391	0.243	0.184	0.147	−0.037	0.124	0.131	0.092	**0.757**
Id.1325	0.261	0.326	0.295	0.059	0.082	0.001	0.242	0.077	0.169	**0.678**
Id.1322	0.369	0.170	0.219	0.308	0.108	0.040	0.171	0.292	0.065	**0.418**

Note: Figures in bold are related to factor loadings greater than 0.40. Extraction Method: Maximum Likelihood. Rotation Method: Varimax with Kaiser Normalization. Rotation converged in 7 iterations.

**Table 3:** Exploratory factor analysis (n = 444)—items related to the psychological domain.

	Factor				
	1	2	3	4	5
Id.1376	**0.701**	0.253	0.138	0.081	0.088
Id.1375	**0.651**	0.178	0.195	0.041	0.175
Id.1377	**0.626**	0.183	0.071	0.207	−0.031
Id.1378	**0.613**	0.124	0.062	0.142	0.282
Id.1379	**0.523**	0.101	0.047	0.162	0.070
Id.1373	0.298	**0.838**	0.131	0.179	−0.008
Id.1374	0.218	**0.738**	0.222	0.069	0.155
Id.1372	0.289	**0.641**	0.117	0.255	0.071
Id.1354	0.099	0.065	**0.784**	0.139	0.219
Id.1353	0.107	0.265	**0.634**	0.234	0.023
Id.1355	0.080	0.076	**0.542**	0.064	0.217
Id.1359	0.193	0.190	**0.521**	0.354	0.111
Id.1363	0.159	0.148	0.165	**0.867**	0.079
Id.1364	0.272	0.075	0.178	**0.704**	0.036
Id.1365	0.110	0.296	0.213	**0.488**	0.092
Id.1370	0.201	0.132	0.227	0.156	**0.880**
Id.1369	0.209	0.040	0.381	0.015	**0.654**

Note: Figures in bold are related to factor loadings greater than 0.40. Extraction Method: Maximum Likelihood. Rotation Method: Varimax with Kaiser Normalization. Rotation converged in 7 iterations.

**Table 4:** Results CFA—Perceptual domain (n = 444)—Assessment of Convergent Validity and Reliability.

Items	Construct	Loadings	AVE	Composite Reliability	Cronbach’s Alpha
Id.1312	Dealing with accidents and supervisor commitment and leadership	0.634	0.501	0.833	0.822
Id.1313		0.757			
Id.1314		0.782			
Id.1315		0.700			
Id.1319		0.654			
Id.1308	Employee commitment	0.858	0.676	0.860	0.845
Id.1309		0.938			
Id.1310		0.643			
Id.1339	Supporting environment: addressing inconveniences	0.759	0.497	0.662	0.658
Id.1340		0.646			
Id.1300	Dealing with near-misses	0.877	0.819	0.900	0.898
Id.1301		0.932			
Id.1316	Management commitment and priority for safety	0.746	0.570	0.799	0.789
Id.1317		0.782			
Id.1318		0.736			
Id.1302	Victim blaming	0.969	0.895	0.944	0.943
Id.1303		0.922			
Id.1320	Safety department commitment	0.820	0.706	0.828	0.825
Id.1321		0.860			
Id.1330	Supporting environment: safety education	0.720	0.573	0.800	0.784
Id.1331		0.710			
Id.1332		0.835			
Id.1327	Supporting environment: time and people	0.864	0.746	0.854	0.854
Id.1328		0.863			
Id.1322	Commitment towards external parties and commitment from external parties	0.703	0.701	0.874	0.857
Id.1325		0.914			
Id.1326		0.879			

**Table 5:** Results CFA—Perceptual domain (n = 444)—assessment of discriminant validity (comparison of squared average variance extracted and constructs’ correlations).

		(1)	(2)	(3)	(4)	(5)	(6)	(7)	(8)	(9)	(10)
(1)	Dealing with accidents and supervisor commitment and leadership										
(2)	Management commitment and priority for safety	0.805									
(3)	Commitment towards external parties and commitment from external parties	0.628	0.594								
(4)	Employee commitment	0.521	0.455	0.648							
(5)	Supporting environment: safety education	0.635	0.687	0.631	0.452						
(6)	Dealing with near-misses	0.483	0.521	0.474	0.395	0.512					
(7)	Supporting environment: addressing inconveniences	0.647	0.534	0.620	0.391	0.560	0.524				
(8)	Victim blaming	0.294	0.153	0.104	0.209	0.154	0.123	0.171			
(9)	Safety department commitment	0.378	0.530	0.415	0.114	0.561	0.367	0.517	0.016		
(10)	Supporting environment: time and people	0.559	0.500	0.461	0.388	0.431	0.351	0.439	0.244	0.246	
	Squared AVE value	0.708	0.755	0.837	0.822	0.757	0.905	0.705	0.946	0.840	0.864

**Table 6:** Results CFA—Psychological domain (n = 444)—assessment of convergent validity and reliability.

Item	Construct	Loadings	AVE	Composite Reliability	Cronbach’s Alpha
Id.1378	Personal priorities and safety responsibilities	0.675	0.515	0.761	0.758
Id.1376		0.750			
Id.1375		0.726			
Id.1374	Intention for proactive safety behaviour	0.789	0.674	0.860	0.847
Id.1373		0.909			
Id.1372		0.757			
Id.1359	Overall safety knowledge, competence and knowledge, and competence during safety problems	0.727	0.527	0.770	0.769
Id.1354		0.727			
Id.1353		0.724			
Id.1365	Trust in the organisation	0.612	0.578	0.801	0.772
Id.1364		0.772			
Id.1363		0.873			
Id.1370	Intention to behave safely/unsafely (in general and under pressure)	0.873	0.713	0.832	0.831
Id.1369		0.815			

**Table 7:** Results CFA—Psychological domain (n = 444)—assessment of discriminant validity (comparison of squared average variance extracted and constructs’ correlations).

		(1)	(2)	(3)	(4)	(5)
(1)	Personal priorities and safety responsibilities					
(2)	Intention for proactive safety behaviour	0.609				
(3)	Safety knowledge and competence (overall and during safety problems)	0.481	0.506			
(4)	Trust in the organization	0.422	0.471	0.604		
(5)	Intention to behave safely/unsafely (in general and under pressure)	0.547	0.310	0.554	0.333	
	Squared AVE value	0.718	0.821	0.726	0.760	0.844

**Table 8:** Non-observable domains of safety culture and their sub-domains.

Organisational Domain/Perceptual Domain			
Sub-Domain	Definition of Sub-Domain	Relevant References (Step B Figure 3)	Example Questions
Dealing with accidents and Supervisor commitment & leadership *	The extent to which: - the workforce is given information about accidents occurring at the workplace; - solutions are searched to prevent similar accidents happening in the future; - the workforce is involved when accidents are addressed. This does not include personal accident history.The extent to which supervisors find safety important, pay attention to safety, set a good example with regard to safety, and discuss safety with their employees.	[19,42,58,59,60,61,62,63]	After an accident, employees are involved in searching for solutions. Supervisors can be approached with questions and concerns about safety.
Dealing with near-misses *	The extent to which: - the workforce is given information about near-misses occurring at the workplace; - solutions are searched to address near-misses; - the workforce is involved when near-misses are addressed. This does not include personal history of encountering near-misses.	[42,58,64,65]	Employees are given information about near-misses occurring in the workplace.
Victim blaming *	The extent to which a guilty party is sought after an accident or a near-miss.	[42,66,67,68]	A guilty party is always sought after an accident. ^▲^
Management commitment and Priority for safety *	The extent to which management finds safety important, pays attention to and values safety, and sets a good example with regard to safety. The extent to which safety considered a priority, under normal working conditions and in case of deviating working conditions (leading to higher work pressure).	[19,21,42,45,58,59,69,70,71,72]	At *[name of the company]*, management attaches a great deal of importance to the safety of employees. The safety rules are observed, even when work is running behind schedule.
Employee commitment *	The extent to which employees at the organisation work safely or unsafely. Specific circumstances are considered, such as working safely when working alone or without supervision.	[19,21,42,59,72,73,74,75]	Everyone works safely at *[name of the company]*, even when no one else is around.
Involving employees	The extent to which the company involves employees in safety matters, such as encouraging employees to report safety problems and taking into consideration suggestions for safety improvement made by employees.	[21,42,45,58,59,76,77,78]	To improve safety, suggestions for improvement made by employees are taken into consideration.
Safety department commitment *	The extent to which the safety department is accessible and has a visible presence within the company.	[79,80,81]	The safety department is easily accessible in the event of safety questions or concerns.
Impact safety department	The extent to which there is a good relationship between the safety department and the employees, supervisors, and top management. It also entails the extent to which the company consults the safety department, sees the safety department as a partner, and follows its recommendations, as well as the extent of the impact/influence of the safety department within the company.	[69,82,83,84]	The recommendations made by the safety department are observed.
Commitment towards external parties and Commitment from external parties *	The extent to which external parties receive assistance and support from the company to guarantee their safety and the extent to which the company monitors the safety compliance of external parties. The extent to which external parties comply with the safety rules as imposed by the company. Specific circumstances are considered, such as safety compliance when working without supervision.	[85,86,87,88,89,90,91,92]	Contractors/consultants are adequately trained to do their jobs at *[name of the company]* safely. Contractors/consultants observe the safety rules at *[name of the company]*.
Supporting environment: time and people *	The extent to which resources (time, people, and budget) are available to facilitate a safe working environment.	[45,93,94,95]	Employees are given adequate time to do their jobs safely.
Supporting environment: safety education *	The extent to which (adequate) training and education on safety is provided. Specific circumstances are considered, such as safety training and education for new workers.	[19,42,45,58,59,69,71,94,96]	Employees are adequately trained to do their jobs safely.
Supporting environment: addressing inconveniences *	The extent to which the company adequately addresses back and muscle complaints, noise nuisance, odour nuisance, and psycho-social risks.	[45,97,98,99,100]	At *[name of the company]*, back and muscle complaints are addressed adequately.
Supporting environment: safety rules	The extent to which the safety rules (and procedures, instructions, and so on) are clear and non-redundant. This sub-domain also entails monitoring safety rules and consequences in case of non-compliance.	[45,58,72,93,101]	At *[name of the company]*, some of the safety rules are actually unnecessary.
Communication and transparency	The extent to which there is an open and transparent communication about safety within the company. This includes the encouragement of and effective reporting of safety issues, complimenting others in case of safe behaviour, approaching others about unsafe behaviour, and the extent to which people can discuss safety issues with another person.	[19,45,58,72,84,102,103]	*This sub-domain is not a separate one, but is incorporated in the other perceptual sub-domain. However, due to the importance of communication and transparency as regards organisational safety, the construct is mentioned here explicitly.*
**Human domain/Psychological domain**			
**Sub-domain**	**Definition of sub-domain**	**Relevant references (step B Figure 3)**	**Example questions**
Personal priorities and Safety responsibilities *	The extent to which individuals attach importance to a safe workplace, and the extent of the importance that safety is continuously emphasised. It also entails being interested in receiving education or training on safety.The extent to which workers feel responsible for their own safety and the safety of others.	[42,44,45,72,74,104,105,106,107,108]	I am interested in receiving education or training on safety. Safety is not my problem; it is the responsibility of the safety department or management. ^▲^
Intention for proactive safety behaviour *	The extent to which individuals report unsafe situations, and the extent to which they approach others (colleagues, supervisors) when they are working in an unsafe manner.	[45,109,110,111]	If I see a colleague working in an unsafe manner, I talk to the colleague about it.
Overall safety knowledge & competence and Knowledge and competence during safety problems *	The extent to which individuals feel sufficiently educated and trained to work safely, and the extent to which they feel like having sufficient skills and knowledge available regarding safety-related aspects, such as safety rules and relevant contact persons.The extent to which individuals feel sufficiently educated and trained to respond in case of emergencies, and the extent to which they feel like having sufficient skills and knowledge to deal with unsafe situations.	[19,42,44,59,82,112,113,114,115,116,117]	I know who I can approach if I have questions about safety. I know what to do in case of emergency (e.g., fire or accident).
Trust in the organisation *	The extent to which individuals feel safe at the company, and the trust they have in the safety within the company. This sub-domain also entails the assessment of the likelihood of their own involvement in an accident.	[42,44,106,118,119]	I think that I will be involved in an accident at *[name of the company]* at some point. ^▲^
Intention to behave safely/ unsafely and Intention to behave safely/ unsafely under pressure *	The extent to which individuals have the intention to comply with safety rules such as wearing personal protective equipment. This sub-domain also entails the extent to which individuals refuse a task when it seems unsafe.The extent to which individuals do not observe safety rules due to pressure from colleagues, supervisors, or top management.	[42,44,45,58,72,73,120,121,122,123,124,125]	If a task is too unsafe, I refuse to do it. Sometimes I do not observe the safety rules due to pressure from colleagues. ^▲^
*^▲^* *Reverse scored questions*			

* The non-observable sub-domains validated by EFA and CFA are indicated with an asterisk.

## Data Availability

Not applicable.
